# On the Therapeutic Use of Monoclonal Antibodies Against Amyloid Plaques in Older Adults with Down Syndrome: A Narrative Review and Perspective

**DOI:** 10.3390/brainsci14111084

**Published:** 2024-10-29

**Authors:** Alberto C. S. Costa

**Affiliations:** Department of Psychiatry, Case Western Reserve University, Cleveland, OH 44106-6090, USA; alberto.costa@case.edu; Tel.: +1-216-368-6378

**Keywords:** Down syndrome, trisomy 21, Alzheimer’s disease, amyloid hypothesis, anti-amyloid monoclonal antibodies, intellectual disability, bioethics

## Abstract

Down syndrome (DS) is a genetic disorder caused by an extra copy of chromosome 21 (trisomy 21 or T21) and is associated with an increased risk of early-onset Alzheimer’s disease (AD), also known as DS-associated AD (DSAD). Individuals with DS typically develop amyloid neuropathology in their late-thirties to early-forties and the mean age of onset of clinical dementia is approximately 55 years. Recent advances in AD clinical research have focused on monoclonal antibodies (mAbs) targeting amyloid-β (Aβ) plaques as a potential therapeutic approach. Therefore, there has been guarded enthusiasm about using anti-amyloid mAbs in the prevention/treatment of DSAD. This narrative review and perspective explores the current understanding of amyloid pathology in AD and DSAD, the rationale for using anti-amyloid mAbs in the treatment of DSAD, and the challenges and opportunities for research toward the application of this therapeutic strategy to older adults with DS.

## 1. Introduction

Down syndrome (DS) is the phenotypic consequence of the trisomy of chromosome 21 (T21) and affects approximately 1 in 700 births worldwide [1,2,3]. This chromosomal disorder has considerable neurodevelopmental impact and is the most common genetically defined cause of intellectual disability [4,5]. DS is also associated with significant neurodegenerative morbidity. Individuals with DS have a considerably higher risk of being afflicted with Alzheimer’s disease (AD) than those without DS, with nearly all individuals with DS developing AD pathology by age 40 [6,7,8] and the mean age of onset of clinical dementia being approximately 55 years [9,10]. Therefore, the development of pharmacological therapies to counteract the neurodevelopmental and neurodegenerative aspects of DS are major unmet needs.

Amyloid plaques, composed primarily of polymeric aggregates of amyloid β (Aβ) peptides, are a hallmark pathologic feature of both AD and DS-associated dementia (DSAD) [6,7,8,11,12,13,14,15,16]. The amyloid hypothesis or amyloid cascade hypothesis has been a dominant theory in AD research for several decades, and in its simplest form posits that the accumulation of Aβ-peptide aggregates in the brain is the primary cause of AD [17,18]. Accordingly, anti-Aβ monoclonal antibodies (mAbs) have emerged as a promising therapeutic approach for reducing amyloid burden in AD [19,20,21,22,23]. These antibodies specifically target and bind to Aβ peptide polymers in various stages of maturation toward the formation of amyloid plaques and promote their clearance via various mechanisms, including phagocytosis by microglia and enhanced transport across the blood–brain barrier [24]. 

On 7 June 2021, aducanumab was approved in a controversial decision by the United States Food and Drug Administration (FDA) [25], making it the first anti-amyloid mAb to be commercialized for patient use anywhere. In January 2024, Biogen announced that it would discontinue the commercialization of this drug for financial reasons, which included dismal sales and a lack of Medicare coverage [26]. Since then, two other anti-amyloid mAbs have received FDA approval for patients with mild cognitive impairment (MCI) or mild AD dementia: lecanemab [27] and donanemab [28]. 

In spite of its popularity and the hundreds of millions of dollars invested to test its validity, the amyloid hypothesis has also faced significant criticism and scrutiny over time. Key criticisms of the amyloid hypothesis include the following: (1) The fact that clinical trials targeting Aβ accumulation have failed to demonstrate significant benefits in terms of clinically relevant cognitive improvement or disease modification [27,28,29,30,31]; (2) The poor correlation between the extent of amyloid deposition and the severity of cognitive decline in patients with AD [29,32,33]; (3) The finding of amyloid plaques in the brains of a significant number of cognitively normal older adults, indicating that amyloid deposition alone may not be sufficient to cause AD [34,35]; (4) The fact that the physiological role of Aβ peptides in the brain is not fully understood [36,37]; and (5) The identification of other compelling potential pathophysiologic mechanisms for AD, such as tau protein tangles, neuroinflammation, synaptic dysfunction, bioenergetics, autophagy, infectious, and vascular factors [24,34,36,38,39,40,41,42]. 

Given the virtually universal presence of AD pathology in individuals with DS in their late 30’s to early 40’s and the inexorable march toward clinical dementia from that time point until they reach their mid-to-late 50’s, DSAD may seem at first glance to be an ideal indication for anti-amyloid mAb treatment [43]. However, neither of the major clinical trials that led to the approval of lecanemab or donanemab recruited participants with DSAD. Consequently, little specific safety and efficacy data are available for patients with DSAD. Critically, the missing information includes the most appropriate time to start treatment, particularly given the very early onset of AD neuropathology in persons with DS. It is equally important to determine whether the greater propensity of persons with DSAD to develop cerebral amyloid angiopathy (CAA) compared to those with sporadic AD could potentially lead to an even greater frequency of cerebral hemorrhage secondary to anti-amyloid mAb treatment than observed in patients without DS [44]. 

More research is certainly needed to establish a broadly acceptable body of evidence supporting or rejecting the use of anti-amyloid mAb treatment in those with DSAD. Given the risks involved, such research is likely to face its own degree of controversy, including the requirement to provide reasonable answers to many ethical issues. These include, but are not limited to, the use of potentially dangerous experimental therapies in vulnerable populations with intellectual disabilities, informed consent and assent procedures, guardianship issues, the need for multiple MRI sessions, potentially under sedation, and ensuring equitable access to novel expensive treatments. These essential ethical considerations should be carefully addressed in the design and performance of clinical trials of mAbs in participants with DS.

This narrative review and perspective explores the current understanding of amyloid pathology in AD and DSAD, the rationale for using mAbs in the treatment of DSAD, and some of the many challenges and opportunities for research toward the application of this therapeutic strategy to older adults with DS.

## 2. The Amyloid Hypothesis in Alzheimer’s Disease

The disorder named by Emil Kraepelin with the eponym of “Alzheimer’s disease” is the most common neurodegenerative disease, affecting approximately 40 million individuals worldwide and is the fifth leading cause of death globally [11,45]. Since the original published descriptions by Alzheimer and Perusini, the very definition of AD is predicated on the pathologic findings of significant neuronal loss, extracellular amyloid plaques, and intracellular tau fibrillary tangles in those with clinical signs and symptoms of dementia. 

Amyloid plaques are the result of the deposition of Aβ peptides in the brain. Aβ is a 4 kDa polypeptide fragment of the amyloid precursor protein (APP), which is widely produced by brain neurons, vascular and blood cells, and, to a lesser extent, astrocytes [17]. Historically, Aβ and its amino acid sequence were initially reported as a primary constituent of meningovascular polymorphic deposits in postmortem specimens from patients with DSAD [13,16]. The full sequence of parenchymal Aβ plaque cores was found to be identical to these perivascular deposits, except that the Aβ in plaque cores mainly extends to the 42nd residue (Aβ_42_). The *APP* gene was sequenced [46], mapped to chromosome 21 [47], and Aβ was confirmed to be a byproduct of the enzymatic processing of APP [48,49,50,51]. 

In line with the definition of this disorder, the presence of amyloid plaques is considered an obligatory feature for a definitive diagnosis of AD. Unsurprisingly, the idea that Aβ aggregation is the causal factor (and not a co-factor or merely an epiphenomenon) for the development of clinical AD dementia quickly became the dominant hypothesis in the field. If this hypothesis were to be confirmed, one of its logical corollaries would be that therapies that reduce Aβ should have a significant beneficial clinical impact on those with AD [52]. However, three decades of work from hundreds of clinics and research laboratories around the world have yielded only weak support for the amyloid hypothesis, and what has become clear is that dementia associated with AD is a multifactorial and much more complex disorder than suggested by a pure version of the amyloid hypothesis. 

With considerable improvements in imaging and cerebrospinal fluid biomarkers and the recent emergence of reliable plasma biomarkers, the past decade has seen the development of two parallel and conflicting notions. The first derives from the many studies indicating that another pathologic feature of AD, tau fibrillary tangles, is more strongly related to clinically observed cognitive decline in those with AD than Aβ accumulation [53,54]. A second parallel notion, which has gained general acceptance, including from regulatory agencies, is that biomarkers can and should be used as valid surrogate endpoints in clinical trials of potential AD therapeutics, with Aβ positron emission tomography (PET) scans being among the main biomarkers used in AD clinical trials [32,33,55,56]. 

In studies using Aβ PET scans as a secondary efficacy measure, the standardized method for quantifying amyloid plaque deposition in the brain involves the Centiloid scale, which has been developed to address variability in amyloid PET results across different tracers, scanners, and analysis methods. In general, the Centiloid scale ranges from 0 to 100, where 0 represents the average amyloid level in young, cognitively normal individuals, and 100 represents the average level in typical patients with AD [57].

With the five most used Aβ tracers demonstrating high affinity and specificity for fibrillar Aβ found in plaques, an emphasis on quantitative Aβ PET scans puts the focus of many clinical trials on the elimination of plaques. Given the poor correlation between the degree of accumulation of cerebral amyloid plaques and the extent of cognitive decline in those with AD, it is not surprising that the dramatic degree of plaque elimination produced by the leading mAbs in major clinical trials has not been accompanied by comparable degrees of cognitive improvement or even clinically significant reductions in the rate of cognitive decline.

## 3. Monoclonal Antibodies Targeting Amyloid Plaques

Despite the many theoretical and conceptual questions that had been raised on the scientific validity of Aβ-targeting as a therapeutic approach to AD and the failure of over 40 clinical trials of amyloid-lowering compounds, several anti-amyloid mAbs were in advanced stages of the development pipeline in the beginning of the 2020s [29]. At that point in time, it seemed that the search for amyloid-lowering agents had become virtually “too big to fail”. In perhaps the best example of this state of affairs, as previously mentioned, the FDA gave accelerated approval to aducanumab in 2021, a decision based primarily on the drug-induced changes in biomarkers such as amyloid PET, tau PET, and cerebrospinal fluid (CSF) phosphorylated tau^181^ (p-tau181) rather than clinical measures of efficacy [25]. This controversial decision resulted in 10 out of 11 independent FDA advisors voting against aducanumab’s approval, 3 of whom resigned from the advisory panel in protest when FDA approval was granted against their recommendation [26,29].

Of the two aducanumab phase 3 trials, called EMERGE and ENGAGE [55], which were virtually identical, large, randomized controlled trials (RCTs), only EMERGE indicated a small but statistically significant difference between the placebo and the high target dose (10 mg/kg) arms in mean scores of clinical instruments used to assess cognitive decline. These instruments were Clinical Dementia Rating Scale Sum of Boxes (CDR-SB), Mini-Mental State Exam (MMSE), Alzheimer’s Disease Assessment Scale–Cognitive Subscale (ADAS-Cog), and Alzheimer’s Disease Cooperative Study—Activities of Daily Living for Mild Cognitive Impairment (ADCS-MCI-ADL). In addition to the absence of any significant efficacy signal as measured by clinical instruments in the ENGAGE trial, a major issue raised by FDA panelists was a significant increase in the frequency of adverse events (AEs) compared to the placebo group. Chief among those AEs were a dose-dependent increase in MRI findings of amyloid-related imaging abnormalities (ARIAs) in both studies and the multiple “red flags” related to data analysis, including inconsistences between EMERGE and ENGAGE results.

The so-called ARIAs are MRI findings typically subdivided into ARIA-E (E = edema, which includes vasogenic edema and sulcal effusions) and ARIA-H (H = hemorrhages, which includes microhemorrhages and superficial siderosis that in rare cases may be associated with intraparenchymal hemorrhage and intraventricular hemorrhage) [58]. Given that many participants of anti-amyloid mAb trials are now deceased and their brains have undergone autopsies confirming areas of edema or hemorrhage, the term “ARIA” should eventually become an outdated euphemism to describe these actual, potentially serious cerebrovascular AEs.

The conclusion of most FDA panelists was that, considering the available data, the risk/benefit ratio for aducanumab did not justify its approval as a pharmacological therapy for AD. Nevertheless, the FDA decided against this recommendation and approved this agent for mild cognitive impairment or mild AD dementia [25]. In contrast, the European Medicines Agency (EMA) rejected the application of aducanumab for medical use in the European Union in December 2021 [25,26]. Finally, following dismal sales of aducanumab in the USA, Biogen announced in January 2024 that it would discontinue the commercialization of this drug.

While the aducanumab saga was taking place, three trials of two other Aβ-targeting mAbs reached their conclusion. The results of the CLARITY AD trial of lecanemab were announced on 27 September 2022 and were published on 5 January 2023 [32]. The results of the GRADUATE 1 and 2 trials of gantenerumab came out on 13 November 2022 [56]. The report of the CLARITY AD trial, which had been a large multicenter RCT involving 1795 participants with early AD, showed that use of lecanemab for an 18-month period produced a 27% slower deterioration on the primary end point (the CDR-SB) compared to a placebo. In addition, all secondary end points were met with statistically significant results. Finally, the mean Aβ level, as assessed by amyloid PET scans, in the lecanemab arm was reduced by 55.48 centiloids compared to a reduction of 3.64 centiloids in the placebo arm. Indeed, this reduction of Aβ levels resulted in a mean Aβ level below the threshold (defined as 24.1 centiloids) for AD positivity in many trial participants.

In contrast to CLARITY AD, the comparably well-powered and designed GRADUATE 1 and 2 trials indicated that the Aβ-targeting mAb, gantenerumab failed to meet the primary end point of a 20% reduction in clinical disease progression [56]. In addition, the observed Aβ reductions of 21.1–24.1 centiloids at Week 54 and 46.8–57.6 at Week 116 were lower than anticipated.

The use of both lecanemab [32] and gantenerumab [56] was associated with higher incidence of cerebral hemorrhage and edema (the so-called ARIA) compared to the control arms of their respective clinical trials. Of these, lecanemab was associated with fewer ARIAs than gantenerumab, while both lecanemab and gantenerumab were associated with a lower frequency of ARIAs than what was observed in the aducanumab [55] phase 3 trials.

The results of the CLARITY AD trial led to the accelerated approval of lecanemab for medical use by the FDA in January 2023, followed by a full approval in July 2023 [27]. The EMA, however, again ruled against approving this drug [31]. In the United Kingdom, lecanemab was recently approved by the Medicines and Healthcare Products Regulatory Agency, but the National Institute for Health and Care Excellence (NICE) decided that it should not be covered by the National Health Service (NHS) due to its “unfavorable benefit–cost ratio” [30].

The results of CLARITY AD and the subsequent approval of lecanemab generated considerable excitement in the field of AD research. After three decades of searching for the proverbial “car key under the lit portion of the parking lot”, there was reason for optimism. Among the many editorials and review papers that chronicled this success story, a narrative review by Perneczky et al. [19] perfectly encapsulates the zeitgeist: “After years of failed attempts to develop a disease-modifying therapy for Alzheimer’s disease, consistent evidence in support of clinical efficacy was finally presented for a monoclonal antibody targeting the amyloid-β protofibrils. In addition to meeting the primary outcome of slowing clinical disease progression over 18 months, secondary clinical outcomes and Aβ lowering on PET also underpin the positive results of the trial”.

The review by Perneczky et al. [19] also made a reasonable argument for why different Aβ-targeting mAbs would show different levels of efficacy, which was based chiefly on the Aβ aggregation states they were directed against. Whereas aducanumab was primarily designed to target mature Aβ deposits (plaques) and gantenerumab is directed to Aβ oligomers and plaques, lecanemab has higher specificity to Aβ fibrils and protofibrils. In other words, perhaps Aβ fibrils and protofibrils represent the “Goldilocks” target level of Aβ polymerization state toward which mAb-based therapies should be directed. This theory would also explain the failure of other amyloid mAb-based agents to produce significant efficacy signals in clinical trials, such as solanezumab, crenezumab, and bapineuzumab, which were mAbs directed against Aβ monomers or oligomers.

When it all seemed to make sense, in May 2023, Eli Lilly reported the results of the TRAILBLAZER-ALZ 2 trial [33], which was another large (1736 participants with early symptomatic AD) phase 3 multicenter RCT of an anti-amyloid mAb-based therapy, LY3002813, or donanemab. Similar to aducanumab and gantenerumab, this anti-amyloid mAb targets primarily Aβ plaques. In spite of this, donanemab performed as well or even better than lecanemab. TRAILBLAZER-ALZ 2 showed that donanemab could slow the pace of cognitive decline in AD by 35% at 76 weeks, as measured by the integrated Alzheimer Disease Rating Scale (iADRS). In addition, the adjusted mean change between donanemab and placebo was 85 centiloids, with 68% of donanemab-treated participants achieving amyloid-negative status. The so-called ARIAs occurred in 24% of the donanemab-treated group versus 2.1% in the placebo group. In July 2024, similar to lecanemab’s initial FDA approval, donanemab was approved for medical use by the FDA under the fast track, priority review, and breakthrough therapy designations [28].

## 4. The Case Against Anti-Amyloid Monoclonal Antibodies

After the FDA approval of lecanemab and donanemab, it is understandable that the popularity of the amyloid hypothesis has become stronger than ever and has returned to the center stage of preclinical and clinical AD research. However, criticism of this hypothesis has not died out. In many ways, portrayals of anti-amyloid mAb-based therapies as “transformative treatments for AD” by pharmaceutical companies and mass media have kindled a new torrent of critical commentary on the limitations and drawbacks of this therapeutic approach and the amyloid hypothesis in general.

In one of the most comprehensive and straightforward published critical analyses of the current state of the amyloid hypothesis, Kepp et al. [29] presented their “16 cautionary notes” on the work that led to the FDA approval of lecanemab. Most of these well-reasoned observations can also be applied to donanemab, and I would direct the reader to read the authors’ original paper for the complete list and their detailed account of these “cautionary notes”. A brief description of some of the most compelling ones can be found in the next several paragraphs.

A key point mentioned by Kepp et al. [29] is the low correlation between the clinical measures of cognitive decline and the biomarker-measured effects. The simple argument here is, if Aβ aggregation is indeed the primary causal factor of AD, why does the dramatic reduction in amyloid produced by mAb-based therapies (with a considerable number of participants achieving amyloid-negative status in both the lecanemab and donanemab trials) not translate into larger clinically measured effect sizes, or even a complete halting of cognitive decline?

Two other important notes mentioned by these authors relate to the large number of potential participants excluded (59.6%) in the CLARITY AD trial and the two times larger dropout rate in the medication arm vs. the placebo arm. In general, a large number of excluded participants in a clinical trial indicates that the inclusion and exclusion criteria of the trial may be too restrictive and will result in a sample of study participants that poorly reflects the targeted patient population. On the other hand, large dropout rates in the treatment group may be an indicator of hidden AEs that have not been detected by the investigators of the study.

Additionally, Kepp et al. [29] noted that the observed effects sizes of the CLARITY AD trial are approximately three times larger in men than in women, and two times larger in the American cohort than in their European counterparts. Given an increased prevalence of AD in women, the first observation brings into question the efficacy of lecanemab in this all-important group of patients. The differences in effect sizes between the American and European cohort may reflect biases in recruitment between sites or/and actual pharmacogenomic variations that may limit the usefulness of lecanemab in certain racial/ethnic groups.

Kepp et al. [29] also observed that lecanemab’s effects were larger in participants without the apolipoprotein E ε4 (*APOE4*) allele compared to *APOE4* heterozygotes, and larger in *APOE4* heterozygotes than in *APOE4* homozygotes. Additionally, this drug’s effects were smaller in participants who were younger than 65 years old compared to those older than 65 years. Given that the amyloid load is typically larger in *APOE4* carriers than in non-carriers, and that older trial participants were likely to have higher levels of Aβ amyloid deposition than younger ones, it seems strange that a treatment aimed at reducing Aβ would have higher efficacy in those who already have comparatively low levels of these depositions. Although this may constitute an unpalatable realization, a potential justification for this finding is that, even in the early stages of the disease, AD might already be irreversible after Aβ deposition reaches a certain threshold.

As aforementioned, both lecanemab and donanemab were associated with increased incidence of MRI-detected brain hemorrhage and edema compared to placebo. Still, both of these mAb-based therapies resulted in a lower frequency of these AEs than seen in the aducanumab phase 3 trials. It is important to note that, for all three anti-Aβ amyloid mAbs, these so-called ARIAs were often clinically silent or were associated with non-life-threatening signs and symptoms, such as migraine that resolved over 3–4 months of treatment suspension. However, ARIAs have been known to lead to hospitalization and death in severe cases. But even in mild cases, little is known about the long-term consequences of ARIAs on brain health. Interestingly, for all three mAbs, ARIAs were more common among *APOE4* carriers compared to non-carriers. After the death by hemorrhagic stroke of three participants under anticoagulation therapy in the aducanumab phase 3 trials, warnings about the use of mAb-based therapies in those under anticoagulation therapy have been mandated by the FDA.

In addition to the increased incidence of MRI-detected brain hemorrhage and edema, a recent systematic review and meta-analysis, aimed at evaluating brain volume changes in subclasses of anti-Aβ drugs, detected an acceleration of volume change that varied by anti-Aβ drug class [59]. In particular, this study found that anti-Aβ amyloid mAbs were associated with accelerated ventricular enlargement, which was strikingly correlated with the frequency of ARIAs. Additionally, participants with MCI who were treated with anti-Aβ drugs in general were “projected to have a material regression toward brain volumes typical of Alzheimer dementia 8 months earlier than if they were untreated”. The authors of this paper conclude that “[t]hese findings reveal the potential for anti-Aβ therapies to compromise long-term brain health by accelerating brain atrophy and provide new insight into the adverse impact of ARIA”.

A final “cautionary note” by Kepp et al. [29] to be discussed in this section relates to the high financial costs compared to the minimal benefits produced by anti-Aβ amyloid mAb treatments. The original annual medication-only cost of aducanumab therapy was $56,000 per patient per year. Following investigations by the US House of Representatives’ Committee on Oversight and Reform and Committee on Energy and Commerce, in December 2021 Biogen announced a 50% price cut for aducanumab to $28,200 [60]. In light of this history, lecanemab’s initial pricing was set at $26,500 and donanemab’s at $32,000 per patient per year [61,62]. These costs do not take into consideration the cost of the periodic brain MRI scans (typically necessary to monitor the development of the so-called ARIAs) and infusion facilities costs. Given the costs of producing high-quality mAbs, these price tags would be justifiable if the clinical benefits of these treatments were of similar scale. Although both lecanemab and donanemab have produced statistically significant effects in their approval trials, neither treatment has produced what most clinicians would consider a clinically significant improvement in patient condition.

**Figure 1:**
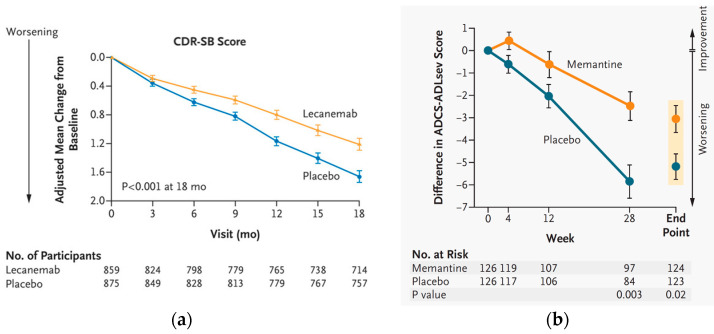
This figure reproduces the primary results of two different trials of AD therapies. (**a**) Adjusted changes in baseline CDR-SB scores in the CLARITY AD trial of the anti-Aβ amyloid mAb lecanemab for the treatment (in orange) and placebo (blue) arms of the study; (**b**) Adjusted changes in baseline ADCS-ADL severity scores in a phase 3 trial of the NMDAR antagonist memantine for the treatment moderate-to-severe AD [64], with the memantine arm appearing as orange and the placebo arm as blue symbols. In all four panels, symbols represent means ± 95% CI.

The reduction in cognitive decline versus placebo for lecanemab and donanemab that were reported in press releases announcing their approval trial results were, respectively, 27% and 36%. These are numbers that can easily be misinterpreted by the lay media and lay public as 27% and 36% improvements in cognitive test scores. Therefore, it is important to consider these numbers in the context of the scales that were actually used in each trial. In absolute numbers, the mean worsening in baseline scores after 18 months of lecanemab treatment was 1.21 for the lecanemab arm and 1.66 for the placebo group (see Figure 1a), i.e., a 0.45 difference in the 18-point CDR-SB scale (whereas a one-point difference in this scale would generally be considered clinically significant). The numbers for donanemab are equally unimpressive. The mean worsening in baseline scores after 76 weeks of treatment in the iADRS scores was −6.02 in the donanemab arm and −9.27 in the placebo group for the low/medium tau study population, and −10.19 in the donanemab arm and −13.11 in the placebo arm in the combined study population (in both study populations, the absolute differences produced by donanemab therapy were approximately 3 points on a 144-point scale; again, statistically significant effects that lack clinical relevance).

Still, the statistically significant effects produced by lecanemab and donanemab in slowing the pace of disease progression have led to the frequent use of the term “disease-modifying” when referring to the effects of these drugs. However, for those old enough to remember the results of the approval trials for donepezil [63] and memantine [64], which were also hypothesized by many to be “disease-modifying” around the time of their respective approvals, it is difficult not to see the parallels with the way the recent results of the anti-Aβ amyloid mAbs have been touted in both the scientific and lay media. To illustrate this point, Figure 1 reproduces the results of approval trials for lecanemab (panels a) together with results of the memantine approval trial (panel b) (unfortunately, at the time of the submission of this paper, the author has not obtained permission from the rightsholders to reproduce published results of the donepezil and donanemab trials).

Although these studies used different efficacy measures and treatment durations (and the treatments were aimed at different disease states), one can see from Figure 1 that both treatments demonstrated a statistically significant reduction in the pace of clinical progression. Indeed (although not shown in this figure), the only AD treatment whose approval clinical trial demonstrated improvement in the primary measure was observed was donepezil [63], even though a later study challenged its cost effectiveness as a long-term therapy for AD [65]. This brings us back to the key question: If amyloid aggregation is indeed the driving pathogenic factor of AD dementia, why does the dramatic reduction in amyloid produced by the new mAb-based therapies not translate into larger clinically measured effect sizes or even complete halting of cognitive decline?

## 5. Amyloid Pathology in Down Syndrome

Brain amyloid plaques in persons with DS typically begin to accumulate in their fourth decade of life. As already mentioned, the APP gene, which encodes the APP protein from which Aβ peptides originate, is located in chromosome 21 and therefore is present in three copies in persons with DS. The consequent overexpression of APP is believed to drive excessive production of Aβ peptides. Indeed, the rare cases of familial AD due to genomic duplication of the APP locus provides clear evidence that an extra copy of this gene is sufficient to produce early-onset AD [66,67,68,69]. In contrast, a single well-documented case of a man with phenotypic DS, partial trisomy of chromosome 21, who lacked triplication of APP and died at 72 years of age without any clinical or pathologic signs of AD, is so-far the strongest evidence that the triplication of this gene is also necessary for the development of early-onset AD in persons with DS [70].

The pathologic hallmarks of DSAD are broadly similar to those historically associated with sporadic AD, i.e., extracellular amyloid plaques, intracellular tau tangles, and significant neuronal cell loss. There are, however, subtle spatiotemporal differences between the pathologic presentation of DSAD and sporadic AD. Temporally, there is a prolonged delay between the age of appearance of amyloid plaques and tau tangles (near-100% prevalence in persons with DS in their mid-to-late 30’s) and the age of onset of clinical dementia (50% prevalence in those with DS in their mid-to-late 50’s). The 20–30-year delay between amyloid positivity and dementia onset in DSAD can be considerably longer than that seen in sporadic AD. In the extreme, the earliest documented case of AD pathology in a person with DS was that of an 8-year-old girl who died of an unrelated cause [7]. This contrasts with case reports of individuals with DS who did not develop clinical dementia into their 60’s, 70’s, and 80’s [71,72,73,74].

The early-onset and prolonged delay between the age of appearance of AD pathology and the age of detection of clinical dementia in persons with DS poses a practical conundrum to clinicians regarding the timing of starting anti-Aβ amyloid mAb-based therapies in those with DS. These drugs have been tested and approved for patients with MCI and mild AD, presumably to more effectively prevent the progression of pathology and avoid severe AEs due to powerful mAb-induced immunological reactivity to widespread amyloid plaques. However, the clinical detection of AD in those with DS tends to happen more than a decade after significant amyloid deposition is already present. When adopting existing adapted criteria for MCI and mild AD in persons with DS, diagnoses of MCI are generally made when patients are between 48 and 56 years of age [74,75,76]. Therefore, it would be reasonable to expect that the relatively late pathology stage for anti-Aβ amyloid mAb-based therapy might be associated with a higher frequency of AEs and serious adverse events (SAEs) in patients with DSAD when compared to those with late-onset sporadic MCI and mild AD. In a perspective paper, Hillerstrom et al. [43] recognized this therapeutic challenge by stating that “[g]iven the extremely high likelihood of early-age amyloid neuropathology in adults with DS, consideration should be given to empirically supported [diagnostic] alternatives that can be more easily obtained, such as validated blood biomarkers”.

Similar to sporadic AD [38,53,54], biomarkers temporally associated with the clinical severity of DSAD are generally more related to the formation of tau tangles than to the severity of extracellular amyloid plaques [77]. Although PET studies have reliably demonstrated a direct correlation of amyloid signal intensity with age in persons with DS, studies investigating a potential relationship between amyloid binding and cognitive performance have yielded uneven results [78,79,80,81,82,83,84,85]. In sporadic AD, positivity on quantitative tau PET, but not Aβ PET or MRI, provided a reliable prediction of conversion from MCI to all-cause dementia when added to a base model including age, sex, education, and MMSE [77]. Another recent study, however, showed that increases in tau were more strongly associated with increases in amyloid for people with DS compared with autosomal-dominant AD [86]. Additionally, these authors found “more rapid onset of tau pathology relative to amyloid plaque presence, as well as a greater magnitude of tau burden relative to amyloid, [which] suggests a shorter overall time course for Alzheimer’s disease pathological progression in people with Down syndrome compared with autosomal-dominant Alzheimer’s disease”. Of the available plasma biomarkers, p-tau217 has shown to be the most accurate blood-based biomarker of both tau and Aβ pathologic brain changes in DS [87,88], whereas neurofilament light chain (NfL) has been found to be a sensitive, but not specific biomarker of neurodegeneration [88,89,90].

Mann et al. [44] found that the severity of plaques formation was significantly greater in those with DS than in cases of sporadic early- or late-onset AD, comparable to what is seen in missense and duplication APP mutations. These authors also found that the severity of CAA was greater in those with DS than in sporadic early- and late-onset AD, but not significantly different than in those with missense and duplication APP mutations. The authors found no significant CAA-type dependence on specific cause of AD. Close inspection of “Figure 5” of that publication, however, indicates that this conclusion may have simply been the result of the authors’ use of the standard classification of CAAs into four types. This classification goes from a more parenchymal and capillary presentation to predominantly vascular phenotypes. This classification could be simplified by combining Types 1 and 2 into what could be considered a mild-to-moderate CAA category, and Types 3 and 4 into a severe CAA category. Then, the combined percentage of Type 3 (where capillary CAA is present along with arterial CAA and few Aβ plaques) and Type 4 CAA (predominantly vascular, in which Aβ deposition is much more prevalent in and around blood vessels), would represent >50% of all CAA in those with DS. If this simpler classification is used, the preferential vascular distribution of plaques in those with DS becomes clear and is second only to that seen in cases of duplication APP, in which the sum of Types 3 and 4 CAA represented 100% of all CAAs.

The higher severity and distinctive spatial distribution of amyloid deposition associated with DSAD (with a preferential vascular distribution and higher prevalence of potentially more clinically consequential CAA subtypes compared to sporadic AD) may pose a significant therapeutic challenge. In a recently published, carefully performed pathologic analysis of tissue from 15 people with DS ranging in age from 43 to 68 years, investigators observed lecanemab-labeled amyloid plaques in all 15 cases, which included extensive binding of lecanemab to brain blood vessels in DS [91]. These authors cautioned that these findings raise “significant safety concerns” and argued for “the necessity for clinical trials of lecanemab in people with DS to evaluate both safety and efficacy, particularly in individuals older than 43 years”.

A different, but related area of concern is the potential for the previously mentioned accelerated brain atrophy observed in a systematic review and meta-analysis of MRIs from individuals who participated in anti-Aβ mAb clinical trials [59]. In that study, the authors considered and discarded the possibility that the “missing volume” could be a byproduct of the clearing of amyloid plaques.

Although the literature emphasizing the amyloid hypothesis can sometimes lead one to imagine that Aβ in persons with AD can occupy considerable brain volume, the reality is that the estimated total mass of Aβ in the brains of those with AD is only around 6.5 mg, compared to 1.7 mg in the brains of cognitively normal controls [92]. This 4.8 mg difference in the amount of Aβ (i.e., the additional amount required for brain Aβ to go beyond the threshold for AD positivity) represents only 0.00032% of the total weight of a typical adult male brain. Another way to visualize what 4.8 mg represents is to note that it amounts to less than a third of the weight of a single grain of long-grain rice (which weighs approximately 16 mg [93]); with rice and brain tissue being of similar densities. There was also a temporal discrepancy between the onset of brain atrophy, which was only detected in various brain regions after 52 weeks of donanemab treatment, and the major (75%) reduction in plaque load that was already observed by 24 weeks posttreatment with this anti-Aβ treatment. Given that this study also found a strong correlation between the occurrence of ARIAs and brain atrophy (involving both grey and white matter), and considering a different report that showed that whole-brain atrophy induced by donanemab [94] was associated with increases in plasma NfL protein, it is likely that the observed brain volume loss reflects neurodegeneration.

A more benign explanation for the brain atrophy observed following anti-Aβ therapies is reduced brain inflammation secondary to plaque removal. Once again, however, the strong correlation between the so-called ARIAs and increases in NfL argue against this possibility. Given that anti-Aβ mAbs are antibodies, which by their very nature trigger downstream immunological reactions, it is more likely that anti-Aβ mAbs would at least transiently trigger neuroinflammation instead of alleviating it. Indeed, it has been shown that activation of Fc gamma receptors (FcγRs), which bind to the constant domain of IgG and are expressed on a wide variety of cell types, including microglia, can result in a pro-inflammatory response including the release of cytokines and other inflammatory mediators [95]. In preclinical models of AD and observations from clinical trials, it has been found that activating FcγRs may be responsible for the activation of microglia following anti-Aβ mAb therapy, which may also be associated with AEs such as ARIAs [95,96].

**Figure 2:**
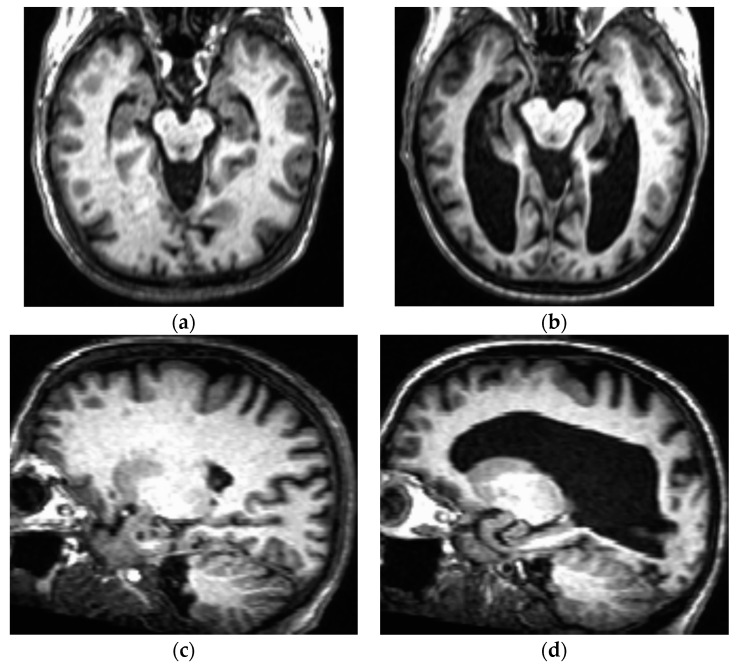
T1-weighted anatomical 3-Tesla MRI images taken from two females with DS, both in the sixth decade of life (generously shared by Dr. Katherine Koenig). One of these individuals (MRIs depicted in panels (**a**,**c**)) did not show signs and symptoms of cognitive decline, whereas the other individual (**b**,**d**) had a formal diagnosis of DSAD at the time of the brain scan. (**a**) Axial section showing expected degrees of neocortical atrophy and lateral ventricular dilation for an older adult with DS; (**b**) Axial section taken at approximately the same anatomical plan as panel a, showing extensive neocortical atrophy and ventriculomegaly indicative of DSAD; (**c**,**d**) Sagittal views from the same individuals taken in the same MRI sessions shown in panels a and b, respectively.

The possibility that anti-Aβ therapy may accelerate brain atrophy is yet another area of concern for the use of this therapeutic class in those with DSAD. This is particularly true because, in general, brains of individuals with DS (even in the absence of AD pathology) are already typically significantly smaller than those of age- and sex-matched peers without DS [97], with a disproportionate volume reductions in the frontal and temporal lobes and cerebellum [98]. In addition, people with DS tend to show accelerated brain aging [99], with their frontal, temporal, and parietal lobes showing greater age-related volume reduction than those of people without DS [100]. Furthermore, brain atrophy associated with DS can be greatly exacerbated by DSAD (see Figure 2 for an example of this phenomenon), with those with DSAD having a 19% lower mean brain weight than those with sporadic AD [101]. Morphometric MRI analyses have associated specific brain regions impacted by DSAD with detectable grey matter changes prior to signs of clinical dementia [102]. These grey matter volume reductions are typically detectable by MRI after amyloid accumulation has already occurred [84], which can actually be considered evidence in favor of the amyloid hypothesis for DSAD.

Besides clearing Aβ, the production of local neuroinflammation by anti-Aβ mAbs might be at the core of the mechanism of action of these agents. For example, recent intervention studies with the innate immune system activator granulocyte-macrophage colony-stimulating factor (GM-CSF; or sargramostim) in animal models of DS, AD, and normal aging have provided preclinical evidence that, during the early stages of AD, activation of the innate immune system may be beneficial [103]. A phase 2 RCT of recombinant human GM-CSF in participants with mild-to-moderate AD showed that subcutaneous injection of sargramostim (5 days/week for 3 weeks) was safe, associated with reduced plasma biomarkers of neuronal damage/neurodegeneration, and improved cognition based on MMSE scores [104]. Therefore, it is not unreasonable to speculate that increased neuroinflammation caused by anti-Aβ mAbs might be one of the reasons behind the statistically significant measured slowdown of cognitive decline seen in the phase 3 clinical trials of these agents.

When analyzing the potential utility and pitfalls of anti-Aβ therapies in both sporadic AD and DSAD, it is important to remember that the physiological role of Aβ peptides in the brain is still not fully understood. There is considerable evidence that, in physiological concentrations, these peptides promote synaptic plasticity and neuronal survival, and that their accumulation in sporadic AD and DSAD may represent dysregulation of normal physiological processes rather than a strictly toxic effect. Therefore, it is not surprising that lowering Aβ using secretase inhibitors has been associated with accelerated cognitive decline and brain atrophy of whole brain and hippocampus in some clinical trials [105,106,107]. In this context, the observed brain atrophy following anti-Aβ mAbs might also be a consequence of the inhibition of the neurotrophic function of Aβ peptides produced by these mAbs (although this effect should be considered theoretically unlikely for mAbs with specific high affinity for mature states of amyloid aggregation, such as donanemab).

In mouse models, pharmacological (secretase inhibitors) or genetic (*APP* knockout) manipulations that result in reductions in Aβ peptide activity depress levels of theta-burst-induced long-term potentiation (LTP) and performance in an object recognition test (ORT) [37]. LTP and some other long-lasting forms of synaptic plasticity are thought to be some of the processes underlying learning and memory [108]. ORT is a simple visual recall task that has been successfully adapted for use in numerous species (including mice, humans, and non-human primates) as a translational assessment of declarative memory [109,110]. Although these observations are still in the realm of preclinical evidence, it is possible that the effect of a reduction in the concentration of Aβ peptides to below physiological levels may aggravate synaptic plasticity and cognitive abilities in individuals with DS. This would be particularly problematic, given the already low baseline level of cognitive ability in most individuals with DS. Additionally, studies in mouse models of DS have consistently demonstrated deficits in theta-burst-induced LTP and ORT [111,112]. Therefore, a therapeutic intervention that produces additional impairment in these preclinical physiological and behavioral proxies to the cognitive deficits displayed by the majority of those with DS should be looked at with added caution.

Beyond its physiological role in promoting synaptic plasticity and neuronal survival, recent studies also suggest that Aβ is an ancient, highly conserved effector molecule of innate immunity [113,114], with broad-spectrum antimicrobial properties against bacteria, viruses, and fungi [115,116]. Additionally, there is growing evidence that many infectious agents may play a role in the pathogenesis of AD and other neurodegenerative disorders [117,118,119,120]. People with DS (mainly during childhood and in old age) are generally more susceptible to viral and bacterial infections [121,122,123]. Therefore, it is critically important to find out whether Aβ (including Aβ aggregates) is a necessary protective element against the spread of infectious agents in the central nervous system.

One highly speculative possibility is that amyloid plaques (which were found to contain viruses and bacteria [124,125]) might be the brain equivalent of granulomas that function as a physical containment to prevent the spread of pathogens that the immune system has failed to kill. If this potentially protective role of amyloid plaques proves to be true, one could imagine a situation in which the pace of a slowly evolving neurodegeneration in AD and DSAD could potentially be hastened or complicated by a concomitant widespread encephalitis caused using anti-Aβ therapies aimed at producing drastic reductions in the levels of Aβ. The logical consequence of this scenario would be that pharmacologically reducing the production or increasing the rate of elimination of this polypeptide below a certain threshold would negatively affect the time course of infection in persons with sporadic AD and DSAD. This hypothetical situation would be akin to the brain spread of the polyomavirus JC in progressive multifocal leukoencephalopathy (PML), which is induced by the weakening of the immune system of patients with multiple sclerosis undergoing treatment with certain biologicals [126].

## 6. Ethical and Practical Considerations

Ethical issues surrounding the use of experimental therapies in vulnerable populations, including individuals with intellectual disabilities, require careful consideration. Informed consent procedures, guardianship issues, and ensuring equitable access to novel treatments are essential ethical considerations that must be addressed in clinical trials and healthcare policies.

There are five neuroethical principles that should guide the conception and operationalization of clinical trials involving agents that affect the central nervous system [127,128]: (1) autonomy and dignity of clinical trial participants; (2) identity preservation; (3) reversibility of treatment; (4) beneficence; (5) and non-maleficence.

Autonomy and dignity of clinical trial participants is an issue of particular importance when it comes to individuals with intellectual disability because of their assumed reduced capacity to provide fully informed consent to undergo the treatment(s) and procedures. The active engagement of caregivers/legal guardians is critical in the consenting process, but equally important is the design of an assent procedure that is catered to the needs of the specific participant population as well as individual needs of each participant. In many cases of DSAD, however, it is likely that the individual’s autonomy may be already so compromised that even the best designed assenting procedures will have little chance of assessing the participant’s true intent. In addition, when facing the accelerated and inexorable march of DSAD-related functional decline, many caregivers/legal guardians may simply be too eager to consent to anything that may provide some benefit, without fully considering potential risks and practical hurdles associated with trial participation. For example, in trials involving individuals with DSAD, it will be essential to emphasize the time and physical requirements of necessary procedures, which can present real challenges for those with DS complicated by behavioral/psychiatric issues (frequently exacerbated by AD). For example, each mAb infusion session takes between 30 min and one hour, and the required ARIA-monitoring periodic MRI scans may require sedation.

It is also important to keep in mind that respect for dignity also includes “dignity of risk”. Dignity of risk is a concept that was born from the disability movement and acknowledges that all life experiences come with an element of risk, which even the best planning and support cannot eliminate [129]. Dignity of risk is seen as closely related to the basic right of self-determination and should be seen as a counterbalance to frequent paternalistic biases of health, educational, and rehabilitation professionals. Historically, such paternalistic attitudes have led to the exclusion of people with intellectual disabilities from research practices [130].

Given that the brain is at the very center of one’s personality, memory, and other key cognitive processes, in practical terms, the issue of identity preservation is always a delicate one to address in neurological and psychiatric interventions and often must be balanced with the other principles [128]. For example, the principle of reversibility of treatment can be seen as closely related to identity preservation in the sense that, whenever identity cannot be completely preserved, it is important to consider whether discontinuation of treatment has the potential to bring the participant’s identity back to baseline. In the case of anti-Aβ mAbs, except for the event of an SAE, it is likely that most effects of treatment can be reversed by discontinuing treatment. This would not be true for active anti-Aβ immunizations for example, given the greater chance of lingering long-term effects secondary to the engagement of long-term immunological memory.

Beneficence and non-maleficence are the basic principles that inform estimations of risk-benefit ratios [127] and are also related to the much-debated concept of equipoise. Equipoise, which is generally defined as uncertainty about the relative effects of the treatments being compared in a trial, is frequently referenced as an important ethical standard for the conduct of RCTs and can also be defined and used in different ways by different individuals [131]. For instance, in 2022, following the 40 RCTs of amyloid-lowering therapies in AD that had failed to improve cognition, with many producing worse mean cognitive test scores, Daly et al. [132] made an equipoise-based argument for the ending of human trials of amyloid-lowering therapies in AD. These authors argued that, given the lack of surprise of the scientific community in the face of multiple RCT failures of amyloid-lowering therapies, they no longer met the operational definition of a lack of “genuine uncertainty” intrinsic to the concept of equipoise. The RCTs leading to the approval of lecanemab and donanemab should be seen as evidence against this argument, but given the many pending issues previously discussed in this paper and elsewhere, the evidence of clinical efficacy produced by these RCTs is far from being a “Hail Mary” for the long-term viability of the amyloid hypothesis.

Considering increased potential for ARIAs in persons with DS, uncertainties about the proper disease stage at which to start treatment, and concerns about aggravation of brain atrophy and memory issues, the additional neuroethical principle of small-step progression of clinical trials should also be an important consideration in the design of future clinical trials of anti-amyloid mAbs for DSAD. Minimizing the number of participants in such trials would be one of the best ways to produce much needed evidence of efficacy while minimizing risk.

The recently published pathologic analysis of tissue from 15 people with DS ranging in age from 43 to 68 years that showed extensive binding of lecanemab to brain blood vessels [91] is concerning and should be reason enough to restrict recruitment in any trial of this mAb (or donanemab) in persons with DS to those younger than 43 years of age. However, it is also important to note that neither lecanemab nor donanemab is approved for the treatment of “pre-dementia” states. Therefore, using either of these drugs for several years as a preventative measure can present added risks.

At this point in time, it can be argued that these data alone might warrant a warning label about DSAD (similar to that for *APOE4* homozygotes) in the prescription information for lecanemab and donanemab. Fortunately, more information should be forthcoming, given that clinicians are required to “[a]dvise patients that the Alzheimer’s Network for Treatment and Diagnostics (ALZ-NET) is a voluntary provider-enrolled patient registry that collects information on treatments for AD, including LEQEMBI. Encourage patients to participate in the ALZ-NET registry” [133]. Just to be clear, this would be a warning specific for DSAD. Therefore, this would be a “double warning” for those with DSAD who happen to be *APOE4* homozygotes (who represent a minority of all cases of DSAD [134]).

If new research can demonstrate that persons with DSAD are at no greater risk of AEs and SAEs resulting from anti-Aβ mAbs than those with sporadic AD, it is likely that patients with DSAD would benefit from subcutaneous application of these agents, which is currently under investigation [135]. Furthermore, the idea that donanemab therapy can potentially be interrupted after the amyloid depositions are cleared [136] could make this therapy particularly attractive for those with DSAD.

## 7. Conclusions

Whereas monoclonal antibodies targeting amyloid plaques offer promising potential as a therapeutic approach for managing dementia in older adults with Down syndrome, several specific challenges and shortcomings of these therapies need to be addressed. These include the following: (1) Limited efficacy in advanced amyloid pathology stages likely to be seen in those with Down syndrome and early Alzheimer’s disease (or even Down syndrome and mild cognitive deficit); (2) Heterogeneity in disease progression; (3) Safety concerns related to the higher frequency of clinically relevant cerebral amyloid angiopathy; access and cost considerations; (4) The multifactorial nature of Alzheimer’s pathology; and (5) Additional ethical considerations and other practical barriers to therapy implementation.

Although the results of anti-Aβ mAb therapies in sporadic Alzheimer’s disease have been disappointing, this should not be considered as the final falsification of the amyloid cascade hypothesis. For example, one could argue that adverse events, such as ARIAs, may be masking potential positive effects of anti-Aβ therapies. It is still possible (although not highly probable) that alternative targeted and highly calibrated approaches to counter amyloid aggregation may yet prove to be successful in the prevention of sporadic AD and DSAD. It is also fair to say that, with the currently available data, one cannot totally discount the possibility that monoclonal antibody-based therapies may yet prove to be safe and efficacious in the treatment of Down syndrome-associated Alzheimer’s disease. Therefore, more research is needed to address this possibility and to advance personalized and effective treatments for individuals with Down syndrome and Alzheimer’s dementia.

## Data Availability

Not applicable.

## References

[1] Patterson D., Costa A.C. (2005). Down syndrome and genetics—A case of linked histories. Nat. Rev. Genet..

[2] de Graaf G., Buckley F., Dever J., Skotko B.G. (2017). Estimation of live birth and population prevalence of Down syndrome in nine U.S. states. Am. J. Med. Genet. Part A.

[3] Mai C.T., Isenburg J.L., Canfield M.A., Meyer R.E., Correa A., Alverson C.J., Lupo P.J., Riehle-Colarusso T., Cho S.J., Aggarwal D. (2019). National population-based estimates for major birth defects, 2010–2014. Birth Defects Res..

[4] Carr J., Collins S. (2018). 50 years with Down syndrome: A longitudinal study. J. Appl. Res. Intellect. Disabil..

[5] Basten I.A., Boada R., Taylor H.G., Koenig K., Barrionuevo V.L., Brandao A.C., Costa A.C.S. (2018). On the Design of Broad-Based Neuropsychological Test Batteries to Assess the Cognitive Abilities of Individuals with Down Syndrome in the Context of Clinical Trials. Brain Sci..

[6] Mann D.M., Yates P.O., Marcyniuk B. (1984). Alzheimer’s presenile dementia, senile dementia of Alzheimer type and Down’s syndrome in middle age form an age related continuum of pathological changes. Neuropathol. Appl. Neurobiol..

[7] Leverenz J.B., Raskind M.A. (1998). Early amyloid deposition in the medial temporal lobe of young Down syndrome patients: A regional quantitative analysis. Exp. Neurol..

[8] Zigman W.B., Lott I.T. (2007). Alzheimer’s disease in Down syndrome: Neurobiology and risk. Ment. Retard. Dev. Disabil. Res. Rev..

[9] Sinai A., Mokrysz C., Bernal J., Bohnen I., Bonell S., Courtenay K., Dodd K., Gazizova D., Hassiotis A., Hillier R. (2018). Predictors of Age of Diagnosis and Survival of Alzheimer’s Disease in Down Syndrome. J. Alzheimer’s Dis..

[10] Rubenstein E., Tewolde S., Michals A., Weuve J., Fortea J., Fox M.P., Pescador Jimenez M., Scott A., Tripodis Y., Skotko B.G. (2024). Alzheimer Dementia among Individuals With Down Syndrome. JAMA Netw. Open.

[11] Cipriani G., Dolciotti C., Picchi L., Bonuccelli U. (2011). Alzheimer and his disease: A brief history. Neurol. Sci..

[12] Hardy J. (2006). Alzheimer’s disease: The amyloid cascade hypothesis: An update and reappraisal. J. Alzheimer’s Dis..

[13] Masters C.L., Simms G., Weinman N.A., Multhaup G., McDonald B.L., Beyreuther K. (1985). Amyloid plaque core protein in Alzheimer disease and Down syndrome. Proc. Natl. Acad. Sci. USA.

[14] Morris G.P., Clark I.A., Vissel B. (2018). Questions concerning the role of amyloid-beta in the definition, aetiology and diagnosis of Alzheimer’s disease. Acta Neuropathol..

[15] Wong C.W., Quaranta V., Glenner G.G. (1985). Neuritic plaques and cerebrovascular amyloid in Alzheimer disease are antigenically related. Proc. Natl. Acad. Sci. USA.

[16] Glenner G.G., Wong C.W. (1984). Alzheimer’s disease and Down’s syndrome: Sharing of a unique cerebrovascular amyloid fibril protein. Biochem. Biophys. Res. Commun..

[17] Hampel H., Hardy J., Blennow K., Chen C., Perry G., Kim S.H., Villemagne V.L., Aisen P., Vendruscolo M., Iwatsubo T. (2021). The Amyloid-beta Pathway in Alzheimer’s Disease. Mol. Psychiatry.

[18] Hardy J., Selkoe D.J. (2002). The amyloid hypothesis of Alzheimer’s disease: Progress and problems on the road to therapeutics. Science.

[19] Perneczky R., Jessen F., Grimmer T., Levin J., Floel A., Peters O., Froelich L. (2023). Anti-amyloid antibody therapies in Alzheimer’s disease. Brain.

[20] Vashisth K., Sharma S., Ghosh S., Babu M.A., Ghosh S., Iqbal D., Kamal M., Almutary A.G., Jha S.K., Ojha S. (2024). Immunotherapy in Alzheimer’s Disease: Current Status and Future Directions. J. Alzheimer’s Dis..

[21] Noorda K., Noorda K., Sabbagh M.N., Bertelson J., Singer J., Decourt B. (2024). Amyloid-Directed Antibodies: Past, Present, and Future. J. Alzheimer’s Dis..

[22] Chhabra A., Solanki S., Saravanabawan P., Venkiteswaran A., Nimmathota N., Modi N.M. (2024). A systematic review of the efficacy and safety of anti-amyloid beta monoclonal antibodies in treatment of Alzheimer’s disease. Expert. Opin. Biol. Ther..

[23] Wang Q., Chen S., Wang J., Shang H., Chen X. (2024). Advancements in Pharmacological Treatment of Alzheimer’s Disease: The Advent of Disease-Modifying Therapies (DMTs). Brain Sci..

[24] Twarowski B., Herbet M. (2023). Inflammatory Processes in Alzheimer’s Disease-Pathomechanism, Diagnosis and Treatment: A Review. Int. J. Mol. Sci..

[25] The Lancet N. (2021). A contentious FDA ruling for Alzheimer’s disease. Lancet Neurol..

[26] Dyer O. (2024). Aduhelm: Biogen abandons Alzheimer’s drug after controversial approval left it unfunded by Medicare. BMJ.

[27] Mahase E. (2023). Alzheimer’s disease: Lecanemab gets full FDA approval and black box safety warning. BMJ.

[28] Lenzer J., Brownlee S. (2024). Donanemab: Conflicts of interest found in FDA committee that approved new Alzheimer’s drug. BMJ.

[29] Kepp K.P., Sensi S.L., Johnsen K.B., Barrio J.R., Hoilund-Carlsen P.F., Neve R.L., Alavi A., Herrup K., Perry G., Robakis N.K. (2023). The Anti-Amyloid Monoclonal Antibody Lecanemab: 16 Cautionary Notes. J. Alzheimer’s Dis..

[30] Kmietowicz Z., Mahase E. (2024). Lecanemab: Benefits of Alzheimer’s drug are “just too small” to justify cost, says NICE. BMJ.

[31] Mahase E. (2024). Lecanemab: European drug agency rejects Alzheimer’s drug amid debate over efficacy and safety. BMJ.

[32] van Dyck C.H., Swanson C.J., Aisen P., Bateman R.J., Chen C., Gee M., Kanekiyo M., Li D., Reyderman L., Cohen S. (2023). Lecanemab in Early Alzheimer’s Disease. N. Engl. J. Med..

[33] Sims J.R., Zimmer J.A., Evans C.D., Lu M., Ardayfio P., Sparks J., Wessels A.M., Shcherbinin S., Wang H., Monkul Nery E.S. (2023). Donanemab in Early Symptomatic Alzheimer Disease: The TRAILBLAZER-ALZ 2 Randomized Clinical Trial. JAMA.

[34] Mecocci P., Boccardi V. (2021). The impact of aging in dementia: It is time to refocus attention on the main risk factor of dementia. Ageing Res. Rev..

[35] Legdeur N., Tijms B.M., Konijnenberg E., den Braber A., Ten Kate M., Sudre C.H., Tomassen J., Badissi M., Yaqub M., Barkhof F. (2020). Associations of Brain Pathology Cognitive and Physical Markers With Age in Cognitively Normal Individuals Aged 60–102 Years. J. Gerontol. A Biol. Sci. Med. Sci..

[36] Puzzo D. (2024). Beyond Amyloid-beta: Reevaluating Its Physiological Role to Find Safe Therapies Against Alzheimer’s Disease. J. Alzheimer’s Dis..

[37] Palmeri A., Ricciarelli R., Gulisano W., Rivera D., Rebosio C., Calcagno E., Tropea M.R., Conti S., Das U., Roy S. (2017). Amyloid-beta Peptide Is Needed for cGMP-Induced Long-Term Potentiation and Memory. J. Neurosci..

[38] Robinson C.G., Lee J., Min P.H., Przybelski S.A., Josephs K.A., Jones D.T., Graff-Radford J., Boeve B.F., Knopman D.S., Jack C.R. (2024). Significance of a positive tau PET scan with a negative amyloid PET scan. Alzheimer’s Dement..

[39] Qu L., Xu S., Lan Z., Fang S., Xu Y., Zhu X. (2024). Apolipoprotein E in Alzheimer’s Disease: Focus on Synaptic Function and Therapeutic Strategy. Mol. Neurobiol..

[40] Frolich L. (2020). Alzheimer’s disease—The ‘microbial hypothesis’ from a clinical and neuroimaging perspective. Psychiatry Res. Neuroimaging.

[41] Patro S., Ratna S., Yamamoto H.A., Ebenezer A.T., Ferguson D.S., Kaur A., McIntyre B.C., Snow R., Solesio M.E. (2021). ATP Synthase and Mitochondrial Bioenergetics Dysfunction in Alzheimer’s Disease. Int. J. Mol. Sci..

[42] Zhang Z., Yang X., Song Y.Q., Tu J. (2021). Autophagy in Alzheimer’s disease pathogenesis: Therapeutic potential and future perspectives. Ageing Res. Rev..

[43] Hillerstrom H., Fisher R., Janicki M.P., Chicoine B., Christian B.T., Esbensen A., Esralew L., Fortea J., Hartley S., Hassenstab J. (2024). Adapting prescribing criteria for amyloid-targeted antibodies for adults with Down syndrome. Alzheimer’s Dement..

[44] Mann D.M.A., Davidson Y.S., Robinson A.C., Allen N., Hashimoto T., Richardson A., Jones M., Snowden J.S., Pendleton N., Potier M.C. (2018). Patterns and severity of vascular amyloid in Alzheimer’s disease associated with duplications and missense mutations in APP gene, Down syndrome and sporadic Alzheimer’s disease. Acta Neuropathol..

[45] (2023). 2023 Alzheimer’s disease facts and figures. Alzheimer’s Dement..

[46] Hattori M., Fujiyama A., Taylor T.D., Watanabe H., Yada T., Park H.S., Toyoda A., Ishii K., Totoki Y., Choi D.K. (2000). The DNA sequence of human chromosome 21. Nature.

[47] Goldgaber D., Lerman M.I., McBride O.W., Saffiotti U., Gajdusek D.C. (1987). Characterization and chromosomal localization of a cDNA encoding brain amyloid of Alzheimer’s disease. Science.

[48] Busciglio J., Gabuzda D.H., Matsudaira P., Yankner B.A. (1993). Generation of beta-amyloid in the secretory pathway in neuronal and nonneuronal cells. Proc. Natl. Acad. Sci. USA.

[49] Haass C., Schlossmacher M.G., Hung A.Y., Vigo-Pelfrey C., Mellon A., Ostaszewski B.L., Lieberburg I., Koo E.H., Schenk D., Teplow D.B. (1992). Amyloid beta-peptide is produced by cultured cells during normal metabolism. Nature.

[50] Shoji M., Golde T.E., Ghiso J., Cheung T.T., Estus S., Shaffer L.M., Cai X.D., McKay D.M., Tintner R., Frangione B. (1992). Production of the Alzheimer amyloid beta protein by normal proteolytic processing. Science.

[51] Seubert P., Vigo-Pelfrey C., Esch F., Lee M., Dovey H., Davis D., Sinha S., Schlossmacher M., Whaley J., Swindlehurst C. (1992). Isolation and quantification of soluble Alzheimer’s beta-peptide from biological fluids. Nature.

[52] Hardy J. (2002). Testing times for the “amyloid cascade hypothesis”. Neurobiol. Aging.

[53] Hanseeuw B.J., Betensky R.A., Jacobs H.I.L., Schultz A.P., Sepulcre J., Becker J.A., Cosio D.M.O., Farrell M., Quiroz Y.T., Mormino E.C. (2019). Association of Amyloid and Tau With Cognition in Preclinical Alzheimer Disease: A Longitudinal Study. JAMA Neurol..

[54] Dubbelman M.A., Mimmack K.J., Sprague E.H., Amariglio R.E., Vannini P., Marshall G.A., Alzheimer’s Disease Neuroimaging I. (2023). Regional cerebral tau predicts decline in everyday functioning across the Alzheimer’s disease spectrum. Alzheimer’s Res. Ther..

[55] Knopman D.S., Jones D.T., Greicius M.D. (2021). Failure to demonstrate efficacy of aducanumab: An analysis of the EMERGE and ENGAGE trials as reported by Biogen, December 2019. Alzheimer’s Dement.

[56] Bateman R.J., Smith J., Donohue M.C., Delmar P., Abbas R., Salloway S., Wojtowicz J., Blennow K., Bittner T., Black S.E. (2023). Two Phase 3 Trials of Gantenerumab in Early Alzheimer’s Disease. N. Engl. J. Med..

[57] Bourgeat P., Dore V., Rowe C.C., Benzinger T., Tosun D., Goyal M.S., LaMontagne P., Jin L., Weiner M.W., Masters C.L. (2023). A universal neocortical mask for Centiloid quantification. Alzheimer’s Dement..

[58] Cogswell P.M., Andrews T.J., Barakos J.A., Barkhof F., Bash S., Benayoun M.D., Chiang G.C., Franceschi A.M., Jack C.R., Pillai J.J. (2024). Alzheimer’s Disease Anti-Amyloid Immunotherapies: Imaging Recommendations and Practice Considerations for ARIA Monitoring. AJNR Am. J. Neuroradiol..

[59] Alves F., Kalinowski P., Ayton S. (2023). Accelerated Brain Volume Loss Caused by Anti-beta-Amyloid Drugs: A Systematic Review and Meta-analysis. Neurology.

[60] Weixel N. (2022). House Investigation Faults FDA Approval of Alzheimer’s Drug Aduhelm as ‘Rife with Irregularities’. The Hill Nexstar.

[61] Lin G., Wright A., Agboola F., Herron-Smith S., Pearson S.D., Rind D.M. (2023). Beta-Amyloid Antibodies for Early Alzheimer’s Disease: Effectiveness and Value.

[62] Belluck P. (2024). New Drug Approved for Early Alzheimer’s. The New York Times.

[63] Rogers S.L., Farlow M.R., Doody R.S., Mohs R., Friedhoff L.T. (1998). A 24-week, double-blind, placebo-controlled trial of donepezil in patients with Alzheimer’s disease. Donepezil Study Group. Neurology.

[64] Reisberg B., Doody R., Stoffler A., Schmitt F., Ferris S., Mobius H.J., Memantine Study Group (2003). Memantine in moderate-to-severe Alzheimer’s disease. N. Engl. J. Med..

[65] Courtney C., Farrell D., Gray R., Hills R., Lynch L., Sellwood E., Edwards S., Hardyman W., Raftery J., Crome P. (2004). Long-term donepezil treatment in 565 patients with Alzheimer’s disease (AD2000): Randomised double-blind trial. Lancet.

[66] Kasuga K., Shimohata T., Nishimura A., Shiga A., Mizuguchi T., Tokunaga J., Ohno T., Miyashita A., Kuwano R., Matsumoto N. (2009). Identification of independent APP locus duplication in Japanese patients with early-onset Alzheimer disease. J. Neurol. Neurosurg. Psychiatry.

[67] Cabrejo L., Guyant-Marechal L., Laquerriere A., Vercelletto M., De la Fourniere F., Thomas-Anterion C., Verny C., Letournel F., Pasquier F., Vital A. (2006). Phenotype associated with APP duplication in five families. Brain.

[68] Rovelet-Lecrux A., Hannequin D., Raux G., Le Meur N., Laquerriere A., Vital A., Dumanchin C., Feuillette S., Brice A., Vercelletto M. (2006). APP locus duplication causes autosomal dominant early-onset Alzheimer disease with cerebral amyloid angiopathy. Nat. Genet..

[69] Sleegers K., Brouwers N., Gijselinck I., Theuns J., Goossens D., Wauters J., Del-Favero J., Cruts M., van Duijn C.M., Van Broeckhoven C. (2006). APP duplication is sufficient to cause early onset Alzheimer’s dementia with cerebral amyloid angiopathy. Brain.

[70] Doran E., Keator D., Head E., Phelan M.J., Kim R., Totoiu M., Barrio J.R., Small G.W., Potkin S.G., Lott I.T. (2017). Down Syndrome, Partial Trisomy 21, and Absence of Alzheimer’s Disease: The Role of APP. J. Alzheimer’s Dis..

[71] Krinsky-McHale S.J., Devenny D.A., Gu H., Jenkins E.C., Kittler P., Murty V.V., Schupf N., Scotto L., Tycko B., Urv T.K. (2008). Successful aging in a 70-year-old man with down syndrome: A case study. Intellect. Dev. Disabil..

[72] Chicoine B., McGuire D. (1997). Longevity of a woman with Down syndrome: A case study. Ment. Retard..

[73] Prasher V.P. (1993). Down’s syndrome, longevity, and Alzheimer’s disease. Br. J. Psychiatry.

[74] Liou J.J., Lou J., Nakagiri J., Yong W., Hom C.L., Doran E.W., Totoiu M., Lott I., Mapstone M., Keator D.B. (2024). A Neuropathology Case Report of a Woman with Down Syndrome who Remained Cognitively Stable. medRxiv.

[75] Burt D.B., Loveland K.A., Primeaux-Hart S., Chen Y.W., Phillips N.B., Cleveland L.A., Lewis K.R., Lesser J., Cummings E. (1998). Dementia in adults with Down syndrome: Diagnostic challenges. Am. J. Ment. Retard..

[76] Devenny D.A., Krinsky-McHale S.J., Sersen G., Silverman W.P. (2000). Sequence of cognitive decline in dementia in adults with Down’s syndrome. J. Intellect. Disabil. Res..

[77] Groot C., Smith R., Collij L.E., Mastenbroek S.E., Stomrud E., Binette A.P., Leuzy A., Palmqvist S., Mattsson-Carlgren N., Strandberg O. (2024). Tau Positron Emission Tomography for Predicting Dementia in Individuals with Mild Cognitive Impairment. JAMA Neurol..

[78] Annus T., Wilson L.R., Hong Y.T., Acosta-Cabronero J., Fryer T.D., Cardenas-Blanco A., Smith R., Boros I., Coles J.P., Aigbirhio F.I. (2016). The pattern of amyloid accumulation in the brains of adults with Down syndrome. Alzheimer’s Dement..

[79] Hartley S.L., Handen B.L., Devenny D.A., Hardison R., Mihaila I., Price J.C., Cohen A.D., Klunk W.E., Mailick M.R., Johnson S.C. (2014). Cognitive functioning in relation to brain amyloid-beta in healthy adults with Down syndrome. Brain.

[80] Nelson L.D., Siddarth P., Kepe V., Scheibel K.E., Huang S.C., Barrio J.R., Small G.W. (2011). Positron emission tomography of brain beta-amyloid and tau levels in adults with Down syndrome. Arch. Neurol..

[81] Rafii M.S., Wishnek H., Brewer J.B., Donohue M.C., Ness S., Mobley W.C., Aisen P.S., Rissman R.A. (2015). The down syndrome biomarker initiative (DSBI) pilot: Proof of concept for deep phenotyping of Alzheimer’s disease biomarkers in down syndrome. Front. Behav. Neurosci..

[82] Jennings D., Seibyl J., Sabbagh M., Lai F., Hopkins W., Bullich S., Gimenez M., Reininger C., Putz B., Stephens A. (2015). Age dependence of brain beta-amyloid deposition in Down syndrome: An [^18^F]florbetaben PET study. Neurology.

[83] Lao P.J., Betthauser T.J., Hillmer A.T., Price J.C., Klunk W.E., Mihaila I., Higgins A.T., Bulova P.D., Hartley S.L., Hardison R. (2016). The effects of normal aging on amyloid-beta deposition in nondemented adults with Down syndrome as imaged by carbon 11-labeled Pittsburgh compound B. Alzheimer’s Dement..

[84] Lao P.J., Handen B.L., Betthauser T.J., Mihaila I., Hartley S.L., Cohen A.D., Tudorascu D.L., Bulova P.D., Lopresti B.J., Tumuluru R.V. (2017). Longitudinal changes in amyloid positron emission tomography and volumetric magnetic resonance imaging in the nondemented Down syndrome population. Alzheimer’s Dement..

[85] Sabbagh M.N., Chen K., Rogers J., Fleisher A.S., Liebsack C., Bandy D., Belden C., Protas H., Thiyyagura P., Liu X. (2015). Florbetapir PET, FDG PET, and MRI in Down syndrome individuals with and without Alzheimer’s dementia. Alzheimer’s Dement..

[86] Wisch J.K., McKay N.S., Boerwinkle A.H., Kennedy J., Flores S., Handen B.L., Christian B.T., Head E., Mapstone M., Rafii M.S. (2024). Comparison of tau spread in people with Down syndrome versus autosomal-dominant Alzheimer’s disease: A cross-sectional study. Lancet Neurol..

[87] Janelidze S., Christian B.T., Price J., Laymon C., Schupf N., Klunk W.E., Lott I., Silverman W., Rosas H.D., Zaman S. (2022). Detection of Brain Tau Pathology in Down Syndrome Using Plasma Biomarkers. JAMA Neurol..

[88] Edwards N.C., Lao P.J., Alshikho M.J., Ericsson O.M., Rizvi B., Petersen M.E., O’Bryant S., Flores-Aguilar L., Simoes S., Mapstone M. (2023). Cerebrovascular disease drives Alzheimer plasma biomarker concentrations in adults with Down syndrome. medRxiv.

[89] Hendrix J.A., Airey D.C., Britton A., Burke A.D., Capone G.T., Chavez R., Chen J., Chicoine B., Costa A.C.S., Dage J.L. (2021). Cross-Sectional Exploration of Plasma Biomarkers of Alzheimer’s Disease in Down Syndrome: Early Data from the Longitudinal Investigation for Enhancing Down Syndrome Research (LIFE-DSR) Study. J. Clin. Med..

[90] Schworer E.K., Handen B.L., Petersen M., O’Bryant S., Peven J.C., Tudorascu D.L., Lee L., Krinsky-McHale S.J., Hom C.L., Clare I.C.H. (2024). Cognitive and functional performance and plasma biomarkers of early Alzheimer’s disease in Down syndrome. Alzheimer’s Dement..

[91] Liu L., Saba A., Pascual J.R., Miller M.B., Hennessey E.L., Lott I.T., Brickman A.M., Wilcock D.M., Harp J.P., Schmitt F.A. (2024). Lecanemab and Vascular-Amyloid Deposition in Brains of People With Down Syndrome. JAMA Neurol..

[92] Roberts B.R., Lind M., Wagen A.Z., Rembach A., Frugier T., Li Q.X., Ryan T.M., McLean C.A., Doecke J.D., Rowe C.C. (2017). Biochemically-defined pools of amyloid-beta in sporadic Alzheimer’s disease: Correlation with amyloid PET. Brain.

[93] Writer S. How Much Does a Single Grain of Rice Weigh?. https://www.reference.com/world-view/much-single-grain-rice-weigh-c39a20469d3fe660.

[94] Pontecorvo M.J., Lu M., Burnham S.C., Schade A.E., Dage J.L., Shcherbinin S., Collins E.C., Sims J.R., Mintun M.A. (2022). Association of Donanemab Treatment With Exploratory Plasma Biomarkers in Early Symptomatic Alzheimer Disease: A Secondary Analysis of the TRAILBLAZER-ALZ Randomized Clinical Trial. JAMA Neurol..

[95] Fuller J.P., Stavenhagen J.B., Teeling J.L. (2014). New roles for Fc receptors in neurodegeneration-the impact on Immunotherapy for Alzheimer’s Disease. Front. Neurosci..

[96] Ramakrishnan V., Friedrich C., Witt C., Sheehan R., Pryor M., Atwal J.K., Wildsmith K., Kudrycki K., Lee S.H., Mazer N. (2023). Quantitative systems pharmacology model of the amyloid pathway in Alzheimer’s disease: Insights into the therapeutic mechanisms of clinical candidates. CPT Pharmacomet. Syst. Pharmacol..

[97] Raz N., Torres I.J., Briggs S.D., Spencer W.D., Thornton A.E., Loken W.J., Gunning F.M., McQuain J.D., Driesen N.R., Acker J.D. (1995). Selective neuroanatomic abnormalities in Down’s syndrome and their cognitive correlates: Evidence from MRI morphometry. Neurology.

[98] Newton R.W., Marder L., Puri S. (2015). Down Syndrome: Current Perspectives.

[99] Cole J.H., Annus T., Wilson L.R., Remtulla R., Hong Y.T., Fryer T.D., Acosta-Cabronero J., Cardenas-Blanco A., Smith R., Menon D.K. (2017). Brain-predicted age in Down syndrome is associated with beta amyloid deposition and cognitive decline. Neurobiol. Aging.

[100] Beacher F., Daly E., Simmons A., Prasher V., Morris R., Robinson C., Lovestone S., Murphy K., Murphy D.G. (2010). Brain anatomy and ageing in non-demented adults with Down’s syndrome: An in vivo MRI study. Psychol. Med..

[101] de la Monte S.M., Hedley-Whyte E.T. (1990). Small cerebral hemispheres in adults with Down’s syndrome: Contributions of developmental arrest and lesions of Alzheimer’s disease. J. Neuropathol. Exp. Neurol..

[102] Matthews D.C., Lukic A.S., Andrews R.D., Marendic B., Brewer J., Rissman R.A., Mosconi L., Strother S.C., Wernick M.N., Mobley W.C. (2016). Dissociation of Down syndrome and Alzheimer’s disease effects with imaging. Alzheimer’s Dement..

[103] Ahmed M.M., Johnson N.R., Boyd T.D., Coughlan C., Chial H.J., Potter H. (2021). Innate Immune System Activation and Neuroinflammation in Down Syndrome and Neurodegeneration: Therapeutic Targets or Partners?. Front. Aging Neurosci..

[104] Potter H., Woodcock J.H., Boyd T.D., Coughlan C.M., O’Shaughnessy J.R., Borges M.T., Thaker A.A., Raj B.A., Adamszuk K., Scott D. (2021). Safety and efficacy of sargramostim (GM-CSF) in the treatment of Alzheimer’s disease. Alzheimer’s Dement..

[105] Egan M.F., Kost J., Tariot P.N., Aisen P.S., Cummings J.L., Vellas B., Sur C., Mukai Y., Voss T., Furtek C. (2018). Randomized Trial of Verubecestat for Mild-to-Moderate Alzheimer’s Disease. N. Engl. J. Med..

[106] Henley D., Raghavan N., Sperling R., Aisen P., Raman R., Romano G. (2019). Preliminary Results of a Trial of Atabecestat in Preclinical Alzheimer’s Disease. N. Engl. J. Med..

[107] Doody R.S., Raman R., Farlow M., Iwatsubo T., Vellas B., Joffe S., Kieburtz K., He F., Sun X., Thomas R.G. (2013). A phase 3 trial of semagacestat for treatment of Alzheimer’s disease. N. Engl. J. Med..

[108] Martin S.J., Grimwood P.D., Morris R.G. (2000). Synaptic plasticity and memory: An evaluation of the hypothesis. Annu. Rev. Neurosci..

[109] Akkerman S., Blokland A., Reneerkens O., van Goethem N.P., Bollen E., Gijselaers H.J., Lieben C.K., Steinbusch H.W., Prickaerts J. (2012). Object recognition testing: Methodological considerations on exploration and discrimination measures. Behav. Brain Res..

[110] Winters B.D., Saksida L.M., Bussey T.J. (2008). Object recognition memory: Neurobiological mechanisms of encoding, consolidation and retrieval. Neurosci. Biobehav. Rev..

[111] Scott-McKean J.J., Roque A.L., Surewicz K., Johnson M.W., Surewicz W.K., Costa A.C.S. (2018). Pharmacological Modulation of Three Modalities of CA1 Hippocampal Long-Term Potentiation in the Ts65Dn Mouse Model of Down Syndrome. Neural Plast..

[112] Fernandez F., Morishita W., Zuniga E., Nguyen J., Blank M., Malenka R.C., Garner C.C. (2007). Pharmacotherapy for cognitive impairment in a mouse model of Down syndrome. Nat. Neurosci..

[113] Gosztyla M.L., Brothers H.M., Robinson S.R. (2018). Alzheimer’s Amyloid-beta is an Antimicrobial Peptide: A Review of the Evidence. J. Alzheimer’s Dis..

[114] Moir R.D., Lathe R., Tanzi R.E. (2018). The antimicrobial protection hypothesis of Alzheimer’s disease. Alzheimer’s Dement..

[115] Soscia S.J., Kirby J.E., Washicosky K.J., Tucker S.M., Ingelsson M., Hyman B., Burton M.A., Goldstein L.E., Duong S., Tanzi R.E. (2010). The Alzheimer’s disease-associated amyloid beta-protein is an antimicrobial peptide. PLoS ONE.

[116] Kumar D.K., Eimer W.A., Tanzi R.E., Moir R.D. (2016). Alzheimer’s disease: The potential therapeutic role of the natural antibiotic amyloid-beta peptide. Neurodegener. Dis. Manag..

[117] Riviere G.R., Riviere K.H., Smith K.S. (2002). Molecular and immunological evidence of oral Treponema in the human brain and their association with Alzheimer’s disease. Oral. Microbiol. Immunol..

[118] Kagan B.L., Jang H., Capone R., Teran Arce F., Ramachandran S., Lal R., Nussinov R. (2012). Antimicrobial properties of amyloid peptides. Mol. Pharm..

[119] Poole S., Singhrao S.K., Kesavalu L., Curtis M.A., Crean S. (2013). Determining the presence of periodontopathic virulence factors in short-term postmortem Alzheimer’s disease brain tissue. J. Alzheimer’s Dis..

[120] Lathe R., Sapronova A., Kotelevtsev Y. (2014). Atherosclerosis and Alzheimer--diseases with a common cause? Inflammation, oxysterols, vasculature. BMC Geriatr..

[121] Verstegen R.H.J., Kusters M.A.A. (2020). Inborn Errors of Adaptive Immunity in Down Syndrome. J. Clin. Immunol..

[122] Emes D., Huls A., Baumer N., Dierssen M., Puri S., Russell L., Sherman S.L., Strydom A., Bargagna S., Brandao A.C. (2021). COVID-19 in Children with Down Syndrome: Data from the Trisomy 21 Research Society Survey. J. Clin. Med..

[123] Huls A., Costa A.C.S., Dierssen M., Baksh R.A., Bargagna S., Baumer N.T., Brandao A.C., Carfi A., Carmona-Iragui M., Chicoine B.A. (2021). Medical vulnerability of individuals with Down syndrome to severe COVID-19-data from the Trisomy 21 Research Society and the UK ISARIC4C survey. EClinicalMedicine.

[124] Wozniak M.A., Mee A.P., Itzhaki R.F. (2009). Herpes simplex virus type 1 DNA is located within Alzheimer’s disease amyloid plaques. J. Pathol..

[125] Miklossy J. (2016). Bacterial Amyloid and DNA are Important Constituents of Senile Plaques: Further Evidence of the Spirochetal and Biofilm Nature of Senile Plaques. J. Alzheimer’s Dis..

[126] Berger J.R. (2010). Progressive multifocal leukoencephalopathy and newer biological agents. Drug Saf..

[127] Schicktanz S., Alpinar-Segawa Z., Ulitsa N., Perry J., Werner P. (2024). Moving Towards Ethical-Practical Recommendations for Alzheimer’s Disease Prediction: Addressing Interindividual, Interprofessional, and Societal Aspects. J. Alzheimer’s Dis..

[128] Jotterand F., Giordano J. (2011). Transcranial magnetic stimulation, deep brain stimulation and personal identity: Ethical questions, and neuroethical approaches for medical practice. Int. Rev. Psychiatry.

[129] Perske R. (1972). The dignity of risk and the mentally retarded. Ment. Retard..

[130] von Benzon N. (2017). Confessions of an inadequate researcher: Space and supervision in research with learning disabled children. Soc. Cult. Geogr..

[131] Dewar B., Chevrier S., De Meulemeester J., Fedyk M., Rodriguez R., Kitto S., Saginur R., Shamy M. (2023). What do we talk about when we talk about “equipoise”? Stakeholder interviews assessing the use of equipoise in clinical research ethics. Trials.

[132] Daly T., Herrup K., Espay A.J. (2024). An Ethical Argument for Ending Human Trials of Amyloid-Lowering Therapies in Alzheimer’s Disease. AJOB Neurosci..

[133] Biogen E.I.A. Treat & Monitor: Initiate Treatment and Monitor for Safety. https://www.leqembihcp.com/treat-and-monitor/initiate-treatment.

[134] Iulita M.F., Bejanin A., Vilaplana E., Carmona-Iragui M., Benejam B., Videla L., Barroeta I., Fernandez S., Altuna M., Pegueroles J. (2023). Association of biological sex with clinical outcomes and biomarkers of Alzheimer’s disease in adults with Down syndrome. Brain Commun..

[135] Park J., Simpson C., Patel K. (2024). Lecanemab: A Humanized Monoclonal Antibody for the Treatment of Early Alzheimer Disease. Ann. Pharmacother..

[136] Shcherbinin S., Evans C.D., Lu M., Andersen S.W., Pontecorvo M.J., Willis B.A., Gueorguieva I., Hauck P.M., Brooks D.A., Mintun M.A. (2022). Association of Amyloid Reduction After Donanemab Treatment With Tau Pathology and Clinical Outcomes: The TRAILBLAZER-ALZ Randomized Clinical Trial. JAMA Neurol..

